# Supplementary material for: Multi-Fold Enhancement of Tocopherol Yields Employing High CO2 Supplementation and Nitrate Limitation in Native Isolate Monoraphidium sp
Source: Cells. 2022 Apr 13;11(8):1315. doi: 10.3390/cells11081315 (PMC9032582; doi:10.3390/cells11081315)
Supplement: Supplementary file 1 [file cells-11-01315-s001.zip › cells-1634160-supplementary.pdf]

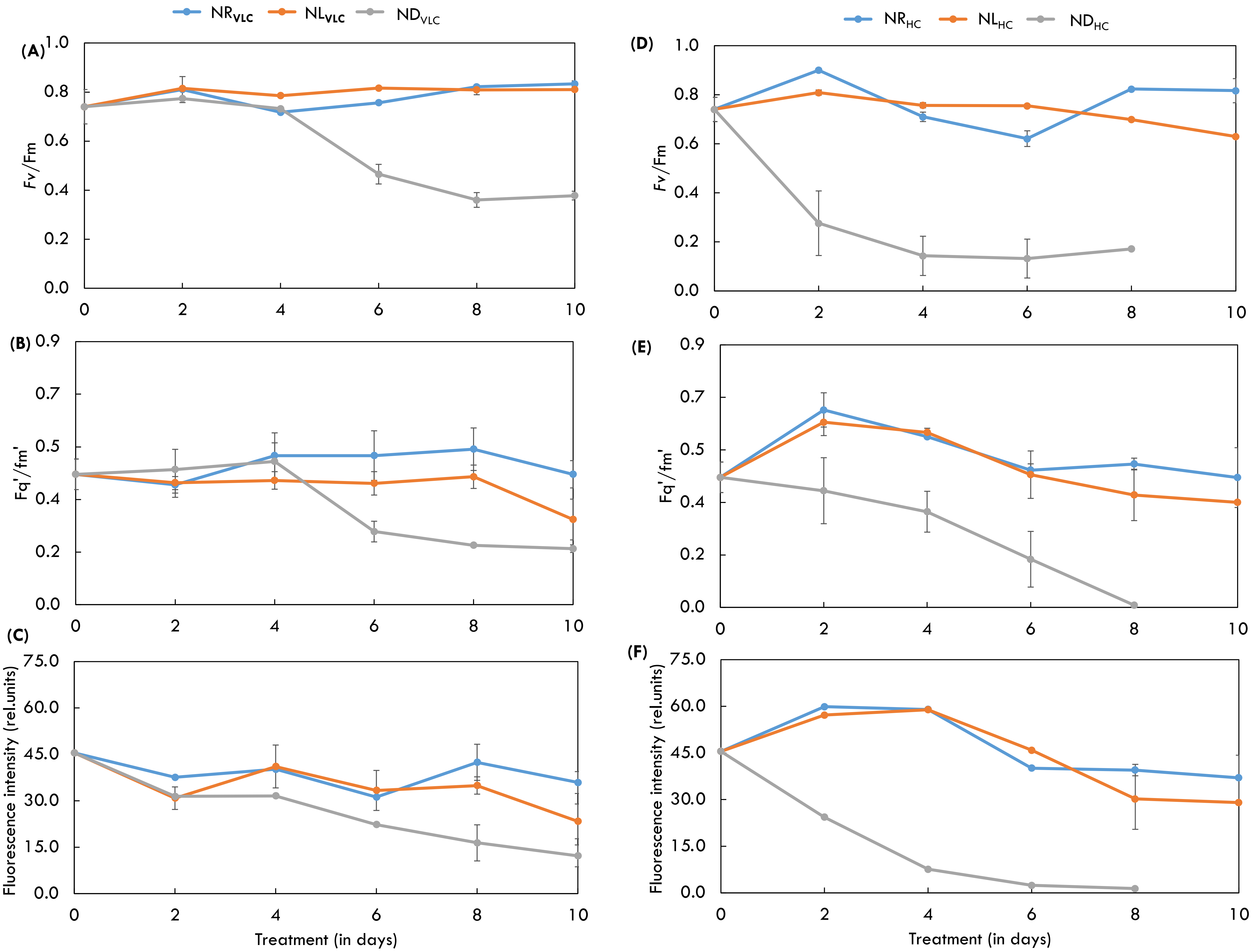

Figure S1: (A & D) Line diagram indicating maximum quantum yields of the PSII efficiency (F_V/F_m) in *Monoraphidium* sp. CABeR41 subjected to NR_{VLC}, NL_{VLC}, ND_{VLC}, NR_{HC}, NL_{HC} and ND_{HC} conditions; **(B & E)** Line diagram indicating PSII operating efficiency i.e., the Fq′/Fm′ ratio; **(C & F)** Changes in electron transport rate (ETR) of the PSII reaction centres

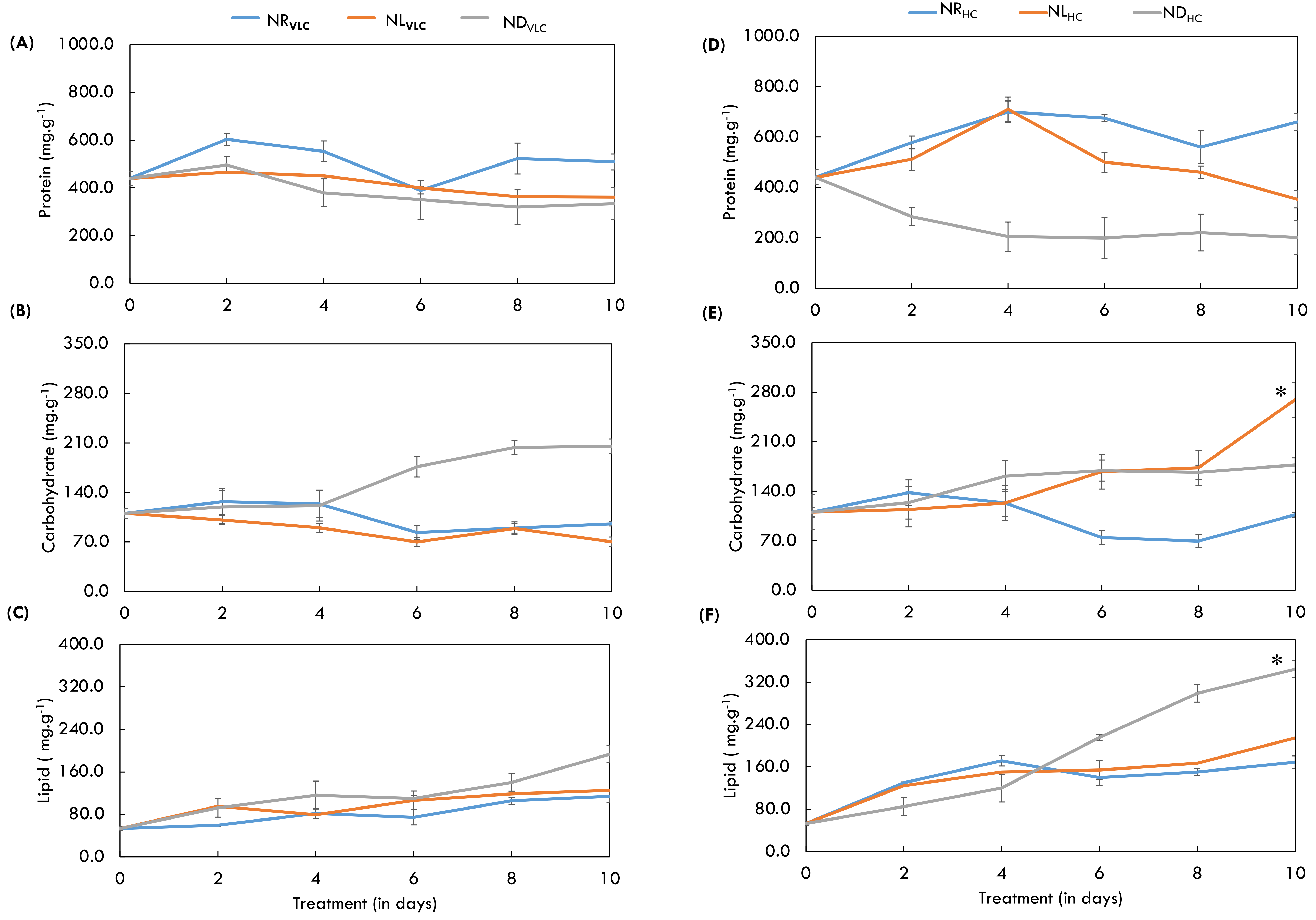

Figure S2: Biochemical profiles of native isolate *Monoraphidium* sp. CABeR41 subjected to NR$_{VLC}$, NL$_{VLC}$, ND$_{VLC}$, NR$_{HC}$, NL$_{HC}$ and ND$_{HC}$ conditions in mg.g^{-1}, **(A & D)** total proteins; **(B & E)** total carbohydrates; and **(C & F)** total lipids (*statistical significance by one-way ANOVA, $P < 0.05$)

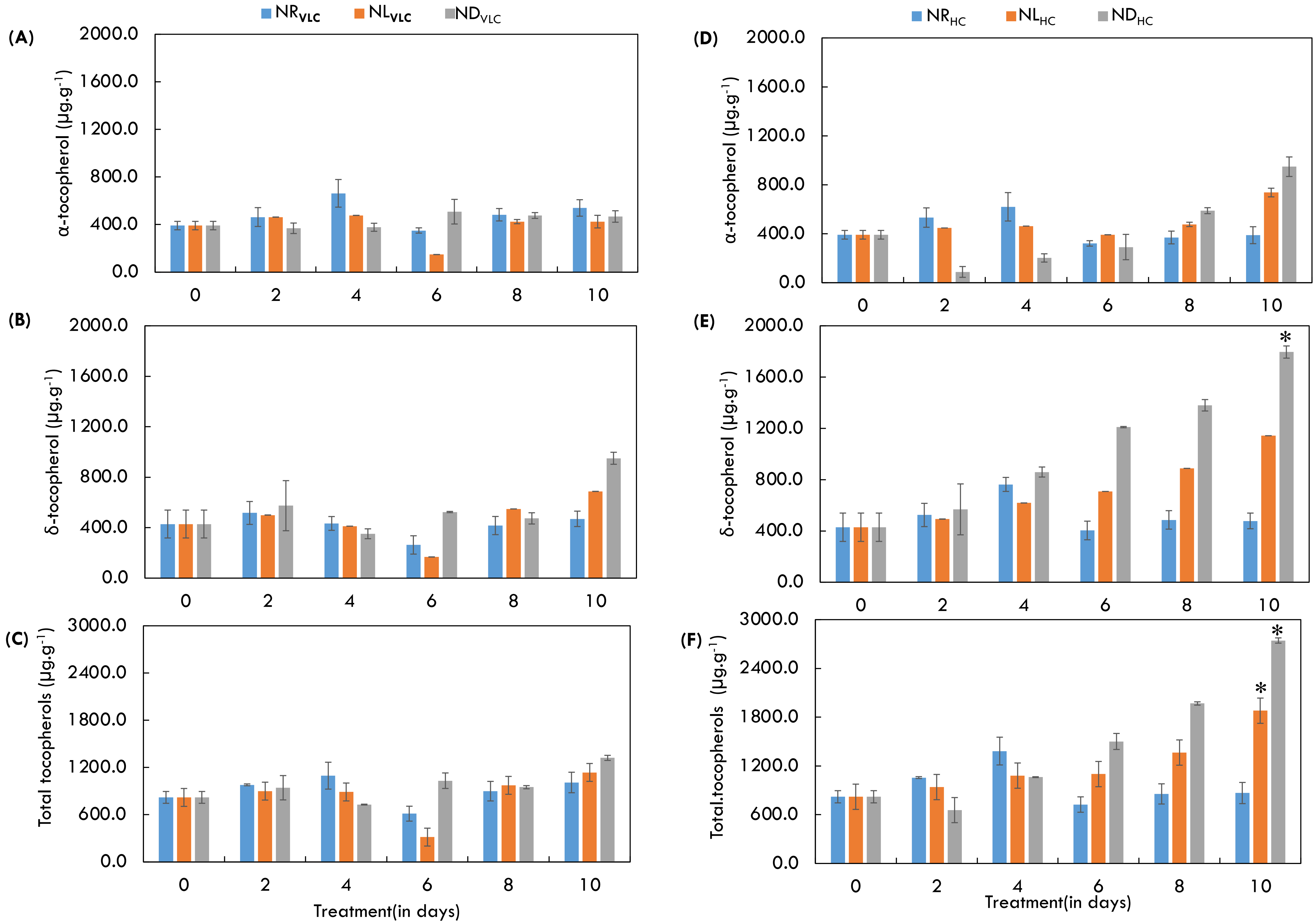

Figure S3: Quantitative analysis of tocopherols (in mg.g^{-1}) of the indigenous strain *Monoraphidium* subjected to NR_{VLC}, NL_{VLC}, ND_{VLC}, NR_{HC}, NL_{HC} and ND_{HC} conditions **(A & D)** *α*-tocopherol; **(B & E)** δ-tocopherol; and **(C & F)** total tocopherols (*statistical significance by one-way ANOVA, $P < 0.05$)

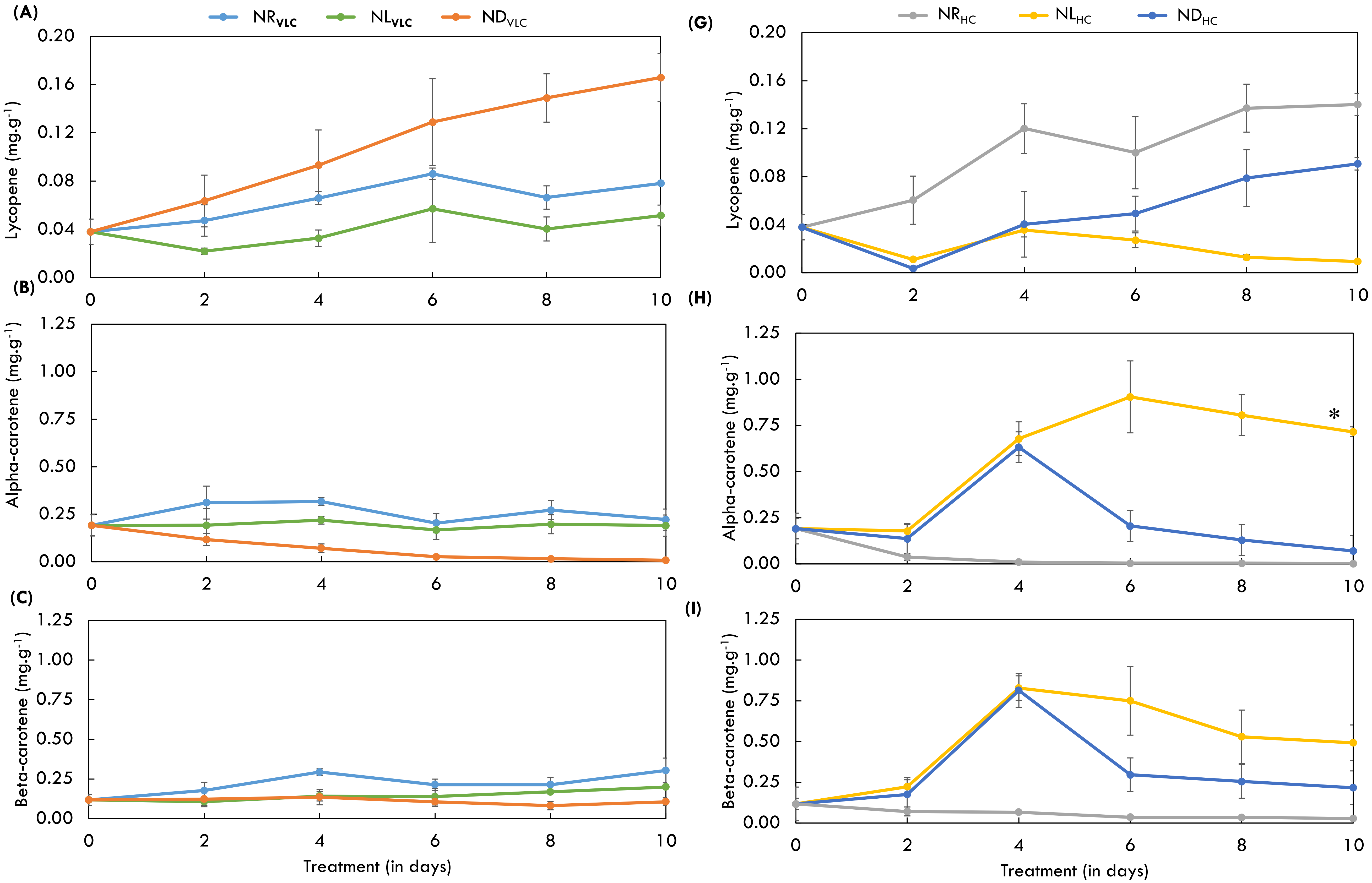

Figure S4: Line diagram representing the time-course profiles of different carotenoids content (mg.g^{-1} dcw) in *Monoraphidium* sp. CABeR41 subjected to NR_{VLC}, NL_{VLC}, ND_{VLC}, NR_{HC}, NL_{HC} and ND_{HC} conditions **(A & G)** Lycopene; **(B & H)** α-carotene; **(C & I)** β-carotene; **(D & J)** Zeaxanthin; **(E & K)** Violaxanthin; **(F & L)** Echinenone (*statistical significance by one-way ANOVA, $P < 0.05$)

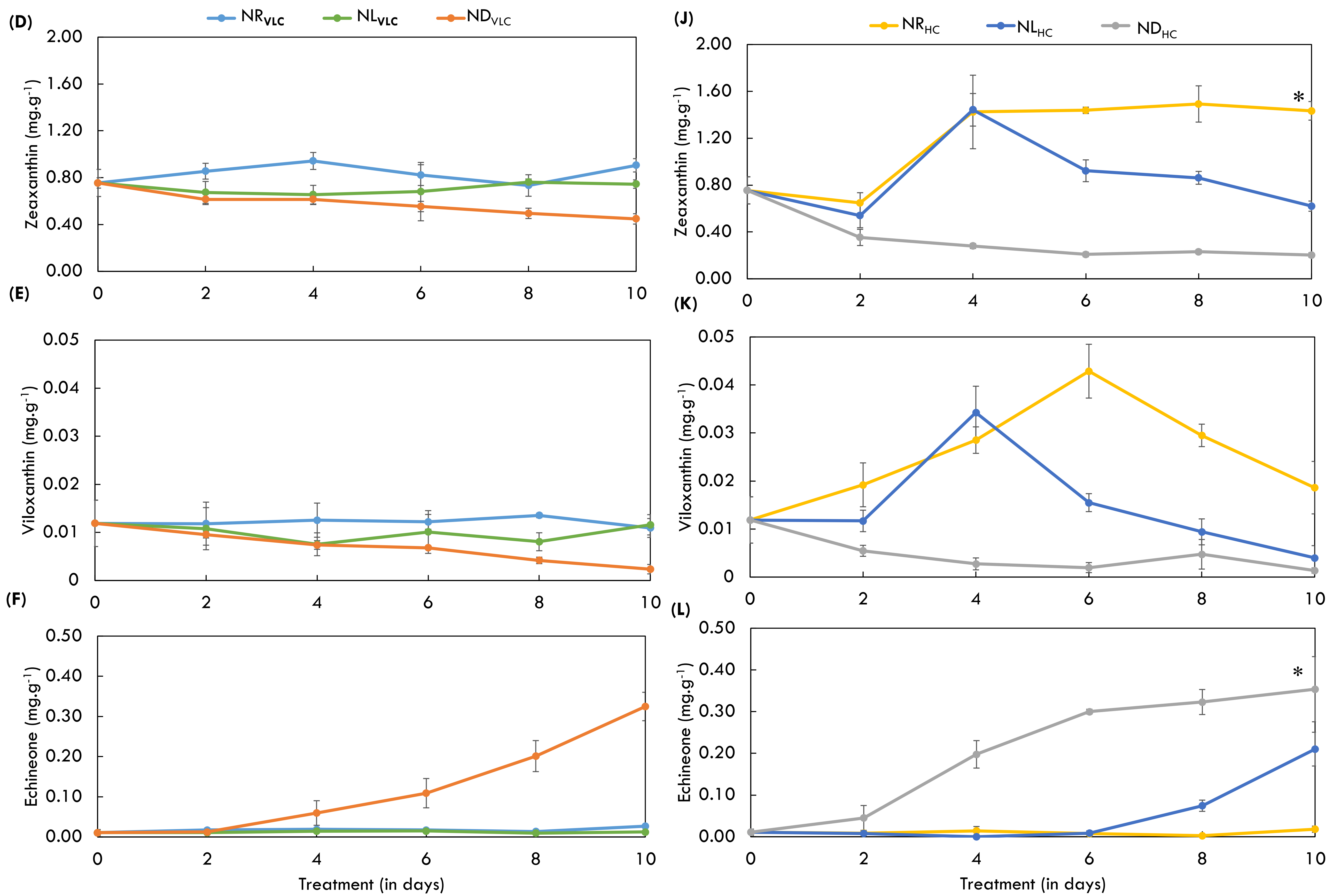

Figure S4: Line diagram representing the time-course profiles of different carotenoids content (mg.g[-1] dcw) in *Monoraphidium* sp. CABeR41 subjected to NR_{VLC}, NL_{VLC}, ND_{VLC}, NR_{HC}, NL_{HC} and ND_{HC} conditions **(A & G)** Lycopene; **(B & H)** α-carotene; **(C & I)** β-carotene; **(D & J)** Zeaxanthin; **(E & K)** Violaxanthin; **(F & L)** Echinenone (*statistical significance by one-way ANOVA, $P < 0.05$)

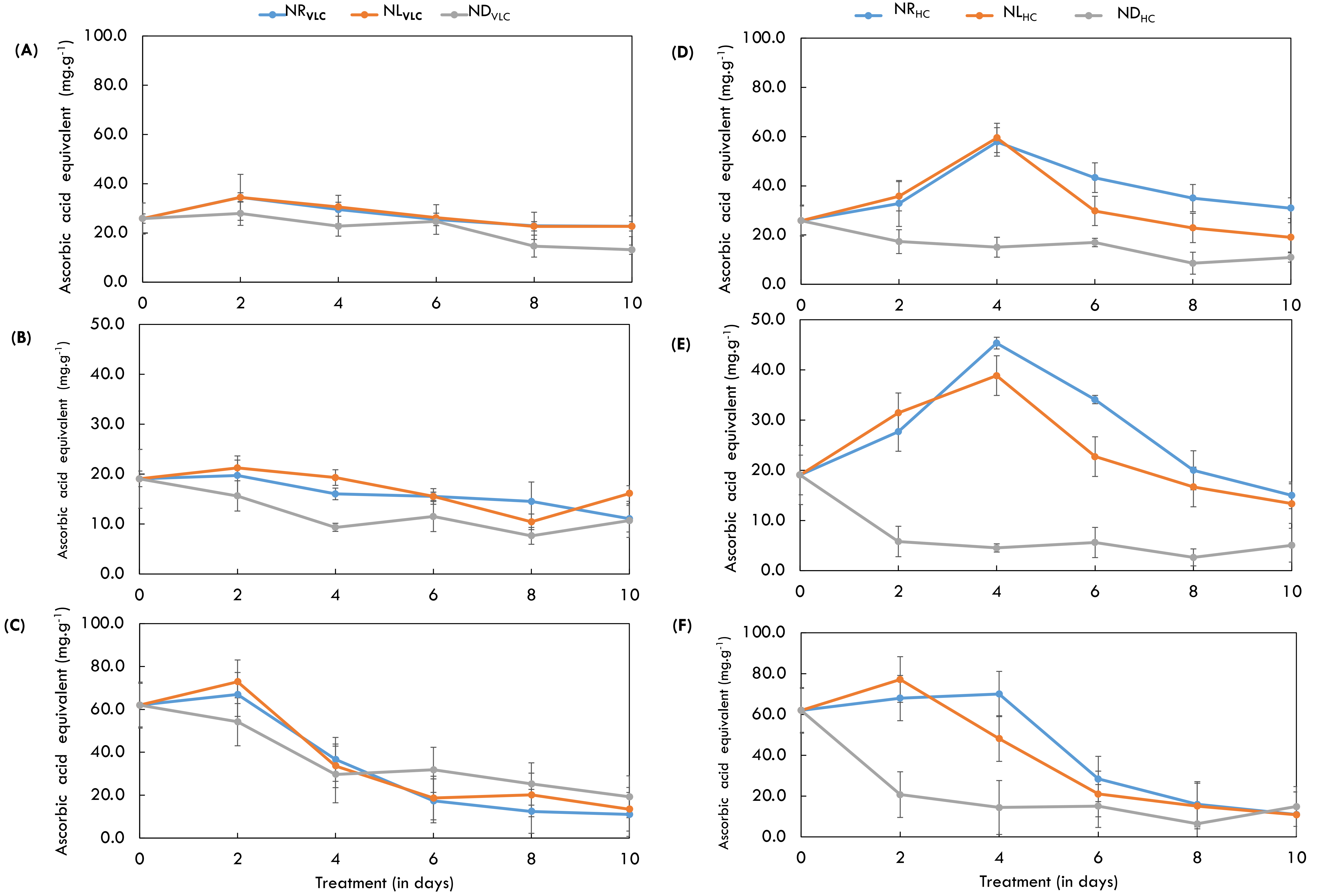

Figure S5: Time-course experiments demonstrating antioxidant efficiency of the microalga *Monoraphidium* sp. CABeR41 subjected to NR$_{VLC}$, NL$_{VLC}$, ND$_{VLC}$, NR$_{HC}$, NL$_{HC}$ and ND$_{HC}$ conditions (in terms of ascorbic acid equivalent mg.g^{-1})

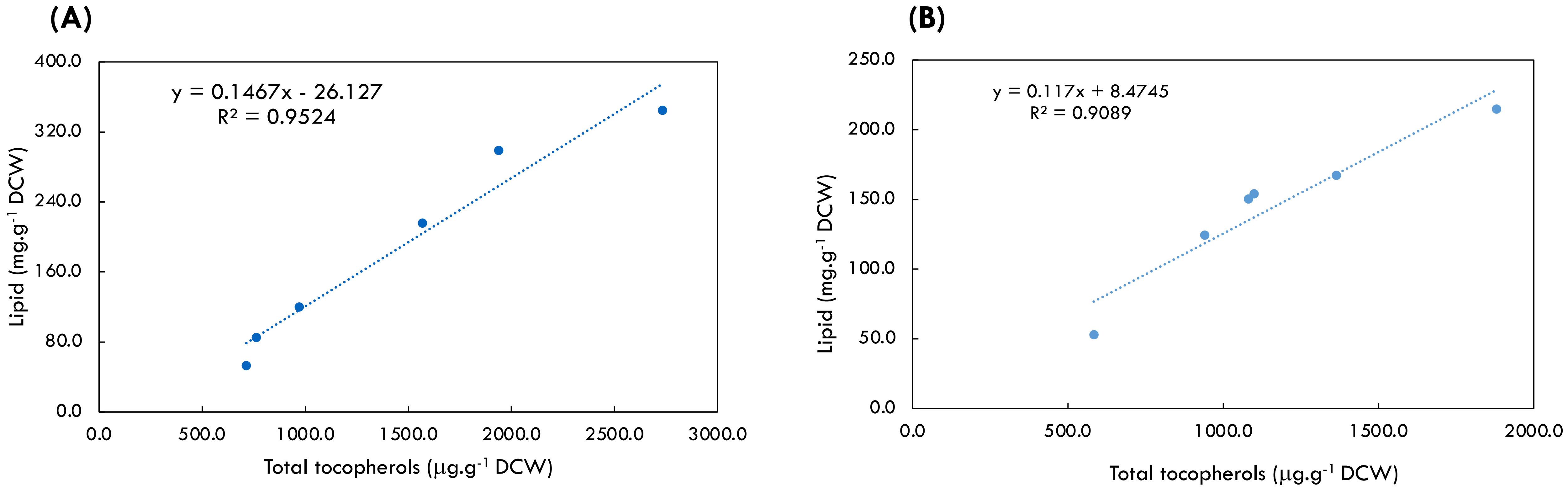

Figure S6: Linear regression analysis depicting the co-relation between total lipids *vs* total tocopherols in native isolate *Monoraphidium* sp. **(A)** ND_{HC} and **(B)** NL_{HC}.
